# Subcellular Evidence for Biogenesis of Autophagosomal Membrane during Spermiogenesis *In vivo*

**DOI:** 10.3389/fphys.2016.00470

**Published:** 2016-10-18

**Authors:** Yufei Huang, Ping Yang, Tengfei Liu, Hong Chen, Xiaoya Chu, Nisar Ahmad, Qian Zhang, Quanfu Li, Lisi Hu, Yi Liu, Qiusheng Chen

**Affiliations:** Laboratory of Animal Cell Biology and Embryology, College of Veterinary Medicine, Nanjing Agricultural UniversityNanjing, China

**Keywords:** isolation membrane, “Chrysanthemum flower center”, endoplasmic reticulum, spermiogenesis, Chinese soft-shelled turtle

## Abstract

Although autophagosome formation has attracted substantial attention, the origin and the source of the autophagosomal membrane remains unresolved. The present study was designed to investigate *in vivo* subcellular evidence for the biogenesis of autophagosomal membrane during spermiogenesis using transmission-electron microscopy (TEM), Western blots and immunohistochemistry in samples from the Chinese soft-shelled turtle. The testis expressed LC3-II protein, which was located within spermatids at different stages of differentiation and indicated active autophagy. TEM showed that numerous autophagosomes were developed inside spermatids. Many endoplasmic reticulum (ER) were transferred into a special “Chrysanthemum flower center” (CFC) in which several double-layer isolation membranes (IM) were formed and extended. The elongated IM always engulfed some cytoplasm and various structures. Narrow tubules connected the ends of multiple ER and the CFC. The CFC was more developed in spermatids with compact nuclei than in spermatids with granular nuclei. An IM could also be transformed from a single ER. Sometimes an IM extended from a trans-Golgi network and wrapped different structures. The plasma membrane of the spermatid invaginated to form vesicles that were distributed among various endosomes around the CFC during spermiogenesis. All this cellular evidence suggests that, *in vivo*, IM was developed mainly by CFC produced from ER within differentiating spermatids during spermiogenesis. Vesicles from Golgi complexes, plasma membranes and endosomes might also be the sources of the autophagosome membrane.

## Introduction

Autophagy is a unique membrane-trafficking process in which newly formed membranes, called phagophores, engulf parts of the cytoplasm and lead to the production of double-membrane autophagosomes that are delivered to lysosomes for degradation(Wild et al., 2011; Rubinsztein et al., 2012). Autophagy is the main cellular process responsible for degrading defective organelles and long-lived proteins. This catabolic pathway has been linked to numerous pathological and physiological conditions (Abada and Elazar, 2014). The physiological role of autophagy was deduced when it was discovered, but the origin of autophagosomal membranes remains unclear(Hamasaki et al., 2013a; Diao et al., 2015).

Autophagy initiates with the emergence of a double-membraned isolation membrane, which encloses organelles and portions of the cytoplasm to form the autophagosome (Hayashi-Nishino et al., 2010; Carlsson and Simonsen, 2015; Sanchez-Wandelmer et al., 2015). Despite much progress in identifying the molecules responsible for autophagosome formation, the origin and the source of the autophagosomal membrane remain unsolved and have been the subject of long-standing debate. Autophagosome formation has recently attracted substantial attention (Abada and Elazar, 2014). Most of the seminal studies on the regulatory mechanisms of autophagosome biogenesis were conducted on *Saccharomyces cerevisiae* or culture cells *in vitro* (Hamasaki and Yoshimori, 2010). The membrane origins of autophagosomes may involve multiple sources (Hayashi-Nishino et al., 2009; Puri et al., 2013), including ER exit sites (ERES) (Zoppino et al., 2010; Graef et al., 2013), the ER–Golgi intermediate compartment (ERGIC) (Ge et al., 2013), the Golgi (Geng et al., 2010; Bodemann et al., 2011), the plasma membrane (Ravikumar et al., 2010) and recycling endosomes (Longatti et al., 2012; Knævelsrud et al., 2013; Puri et al., 2013). However, there is little ultrastructural information from the initial stage of autophagosome formation. It is unclear if and where distinct membrane sources fuse during autophagosome biogenesis.

A spermatozoon is a highly differentiated cell developed from a spermatid through spermiogenesis in the convoluted seminiferous tubule of the testis. Spermiogenesis is the intracellular clearance process for male gametes. The decrease in cell volume occurs due to the shedding of unnecessary cytoplasm and organelles, but the process for reptilian gametes remains largely unknown (Zhang et al., 2007). Our previous studies showed that epididymal spermatozoa of Chinese soft-shelled turtles contain a huge cytoplasmic droplet with numerous lipid droplets that serve as energy and nutrition sources, which favors long-term sperm storage (Zhang et al., 2015). There was not an obvious shedding process of unnecessary cytoplasm during spermiogenesis although, the cell size of the maturing spermatozoon was reduced. The cytoplasmic droplet always attached to the mid-piece of the spermatozoon without migrating down the sperm tail. In the present study, a subcellular mechanism for autophagosomal membrane biogenesis was examined in detail during *in vivo* turtle spermiogenesis. The turtle could be a potential animal model for long-term sperm storage at atmospheric temperature(Chen et al., 2015).

## Materials and methods

### Animals

All procedures with turtles were conducted according to the Animal Research Institute Committee guidelines of Nanjing Agriculture University, China. Fifteen male adult Chinese soft-shelled turtles, aged 4–5 years, were purchased from a wild breeding base in Nanjing, Southeastern China (GPS coordinates N 32.050 E 118.783). The animals were collected in August, September and October. After breeding in the lab for 24 h, the turtles were rendered comatose using intraperitoneal sodium pentobarbital (20 mg/kg) and killed by cervical dislocation. One side of the testis was collected immediately after death and fixed for light and transmission electron microscopy. The other testis was stored at −70°C for Western blot analysis. The sampling procedures were approved by the Nanjing Agricultural University Veterinary College. The protocol was approved by the Science and Technology Agency of Jiangsu Province. The approval ID is SYXK (SU) 2010-0005. All efforts were made to minimize the animal's suffering.

### Western blotting

Samples of the testis in each group were homogenized in ice-cold RIPA buffer (25 mM Tris/HCl (pH 7.6), 150 mM NaCl, 1% sodium deoxycholate, 1% Nonidet-P40, 0.1% SDS, 0.05 mM PMSF), and centrifuged at 15,000 g for 10 min at 4°C. Then, the total protein concentration was determined with a BCA protein assay (Santa Cruz, sc-202389). Samples (40 μg protein per lane) were subjected to electrophoresis on a 10% SDS-PAGE gel and then transferred onto PVDF membranes (Millipore, ISEQ00010). After nonspecific blocking in 5% nonfat milk, the membranes were incubated with an anti-LC3B (1:1000 dilution) antibody (Abcam, ab48394) overnight at 4°C. After washing with TBST, the membranes were incubated with peroxidase-linked goat anti-rabbit IgG (1:5000, Bioworld Technology Inc., BS13278) for 2 h. Following incubation, the bound antibodies were visualized by using the ECL detection system (Vazyme Biotech, E411-04). Immunoreactive bands were quantified with Quantity One software (Bio-Rad Laboratories).

### Immunohistochemistry

In brief, after deparaffinization and hydration, 3% H_2_O_2_ was added to the paraffin sections to eliminate internal peroxidase activity. Then, slides were boiled in buffered citrate. Next, sections were blocked using 5% BSA and then incubated with anti-LC3B (1:200) antibody (Abcam, ab48394) at 4°C overnight. Negative controls were prepared by exchanging the primary antibody with PBS. After washing in PBS, the sections were incubated with goat anti-rabbit IgG (Santa Cruz, sc2004) for 1 h at 37°C. After a brief wash in PBS, peroxidase activity was determined using DAB (Sigma, D8001). Images were collected under a light microscope (Olympus DP73).

### Transmission electron microscopy

The testis was immediately removed and cut into 1 mm^3^ small blocks. After that, we immerse the samples into 2.5% glutaraldehyde fixing agent mixed with phosphate buffered saline at 4°C (PBS, 0.1 M, pH 7.4) overnight. The tissues were rinsed in the same PBS and fixed in 1% buffered osmium tetroxide. Then we washed the samples in the buffer. The testis was dehydrated in ascendant concentrations of ethanol and permeated with a propylene oxide–Araldite compound before they were embedded in Araldite. Under an ultramicrotome, we sectioned the blocks. The sections (50 nm) were stained with 1% uranyl acetate and lead citrate. Finally, slices were examined and photographed using a Hitachi H-7650 TEM.

### Statistical analysis

All data were presented as the means ± SE. The TEM pictures were imported into Image-Pro Plus (IPP) 6.0 software for statistical data analysis. The statistical analysis was performed using SPSS software version 14.0 with one-way analysis of variance (ANOVA). The data were considered statistically significant when *P* < 0.05.

## Results

### The testis expressed LC3-II protein, which was located within spermatids and indicated active autophagy

**Figure 1:**
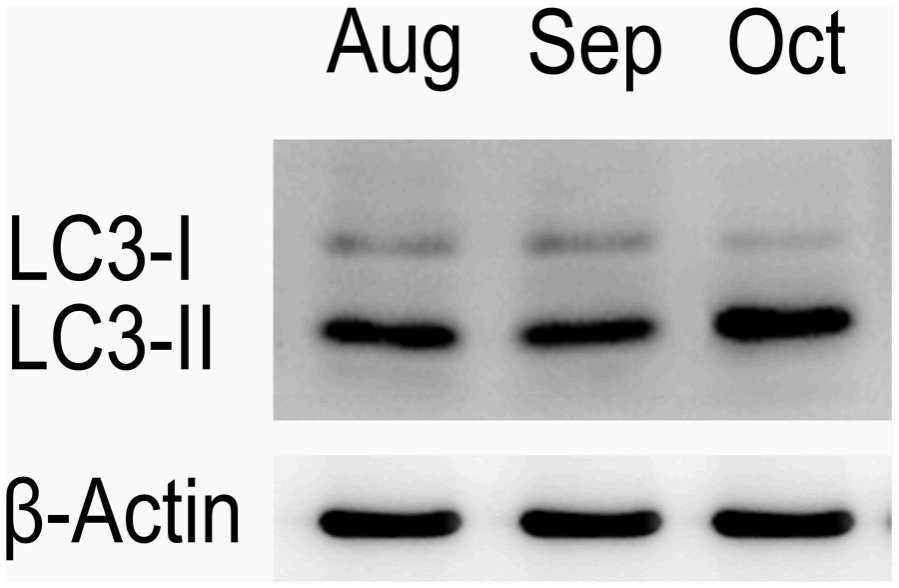
**Western blot analysis of LC3 A protein in the testis of *P. sinensis***.

**Figure 2:**
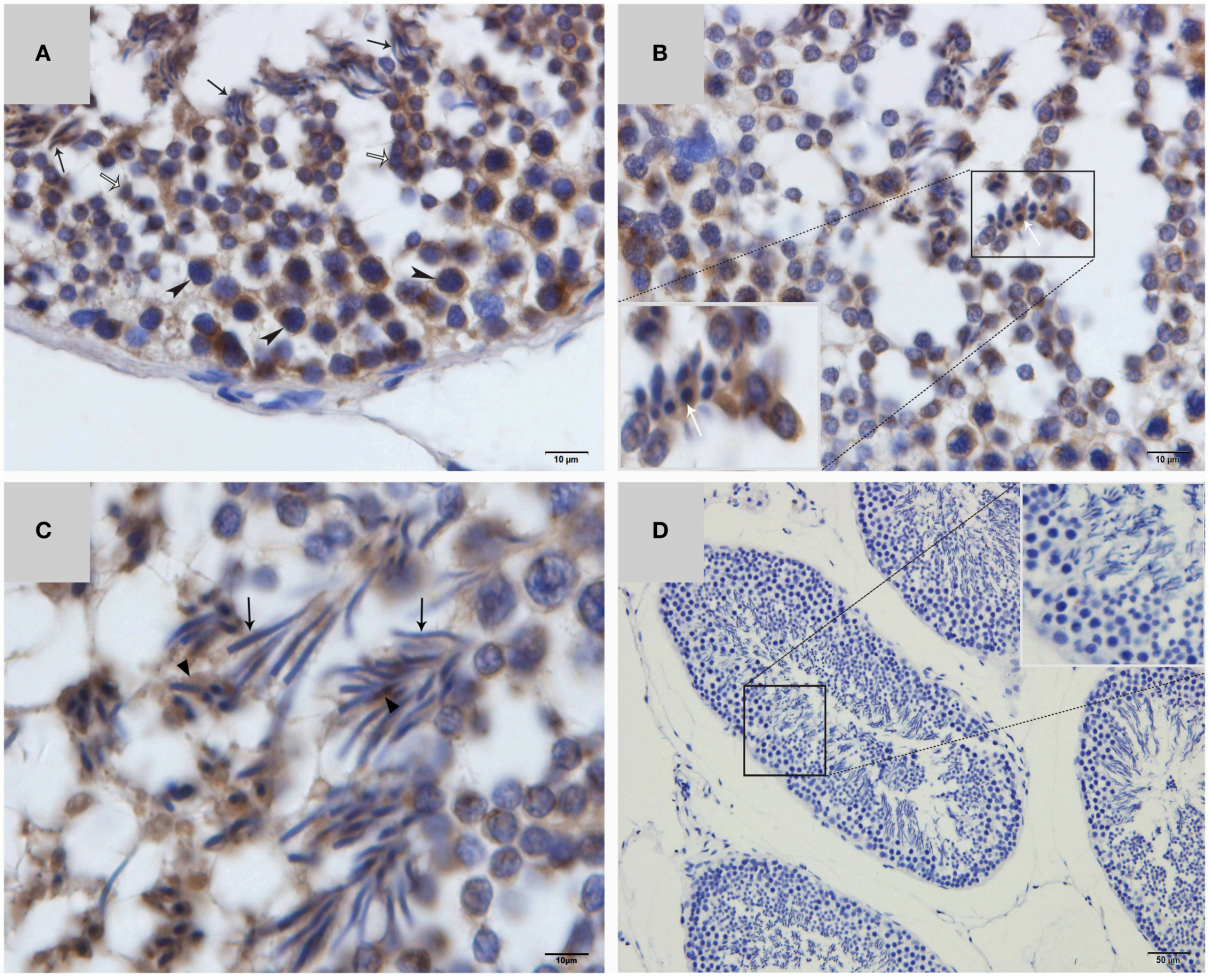
**LC3 immunohistochemistry on ST of turtle testis. (A)** August; **(B)** September; **(C)** October; **(D)** Negative control; Spermatozoa (↑), cytoplasmic droplet (▴), differentiating spermatid (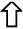), spermatocyte (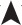).

Western blot tests showed that the turtle testis expressed LC3, which is a specific marker protein for autophagosomes, during spermiogenesis (Figure 1). LC3 immunohistochemistry of seminiferous tubules (ST) further showed that LC3 was distributed in different germ cells (Figure 2), including spermatocytes(Figure 2A), differentiating spermatids (Figure 2B), spermatozoa (Figures 2A,C), and cytoplasmic droplets (Figure 2C).

### IM was developed mainly by CFC produced from endoplasmic reticulum (ER)

**Figure 3:**
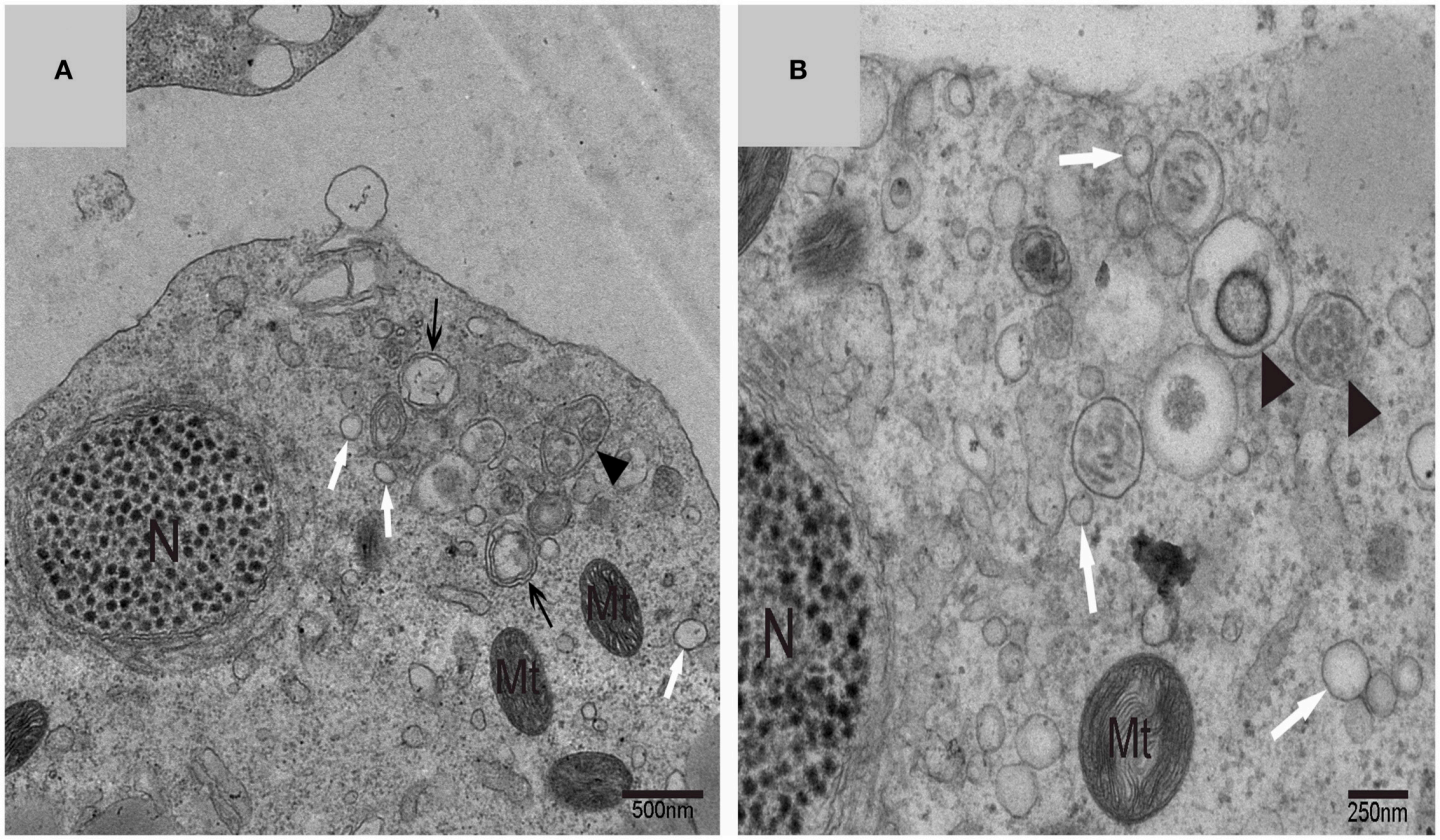
**TEM photograph of a differentiating spermatid**. Granular nucleus (N), autophagosomes (↑), mitochondrion (Mt), multivesicular body (▴), small vesicle (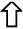). **(A)** Low magnification of a differentiating spermatid; **(B)** high magnification of a differentiating spermatid.

**Figure 4:**
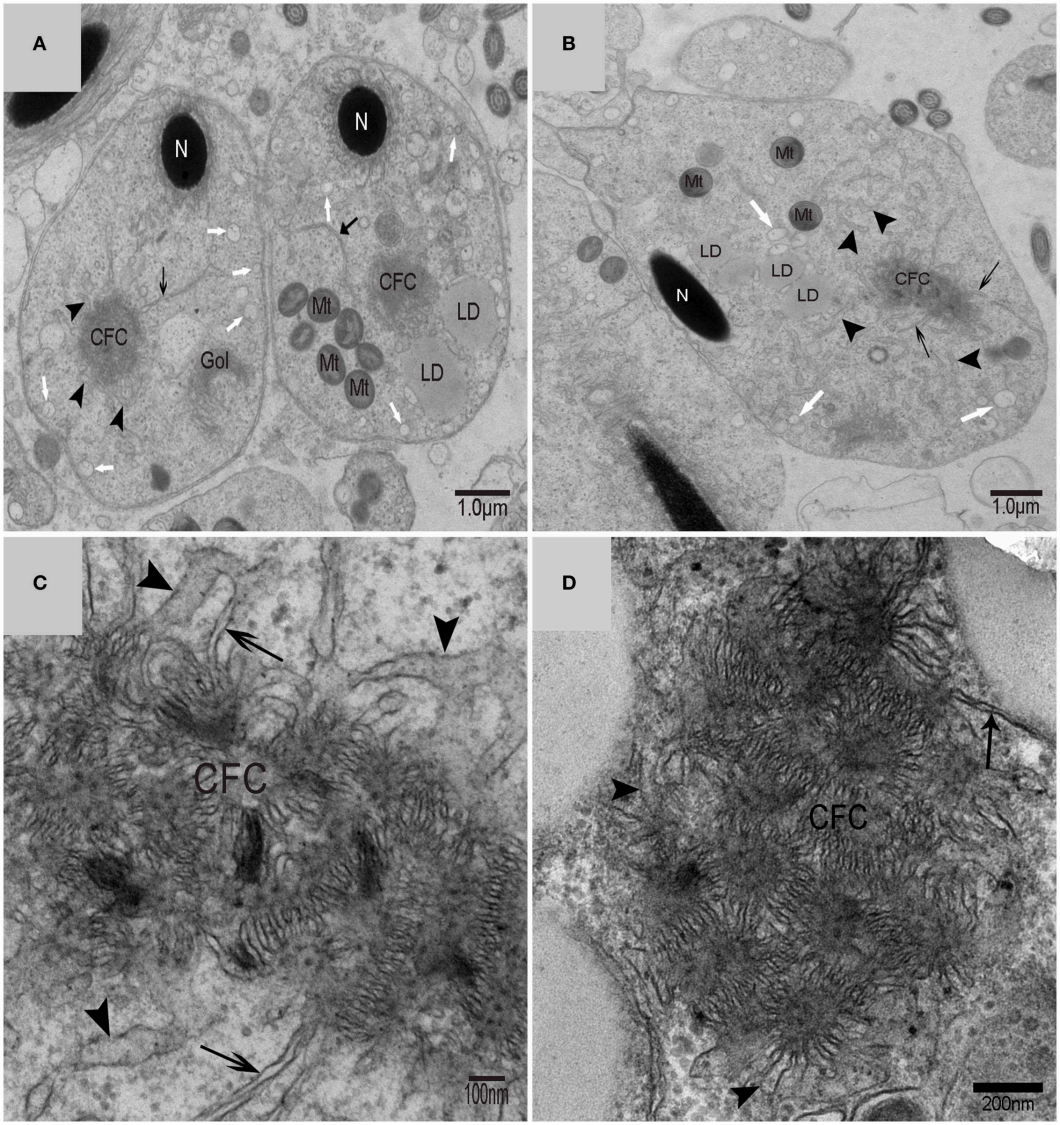
**TEM photograph of the “Chrysanthemum flower center” in the spermatid**. **(A,B)** Low magnification of the spermatids; **(C,D)** high magnification of the “Chrysanthemum flower center.” Compact nucleus (N), Chrysanthemum flower center (CFC), mitochondrion (Mt), Golgi complex (Gol), lipid droplet (LD), endoplasmic reticulum (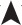), vesicle (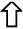), isolation membrane (↑), wrapping autophagosome (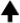).

**Figure 5:**
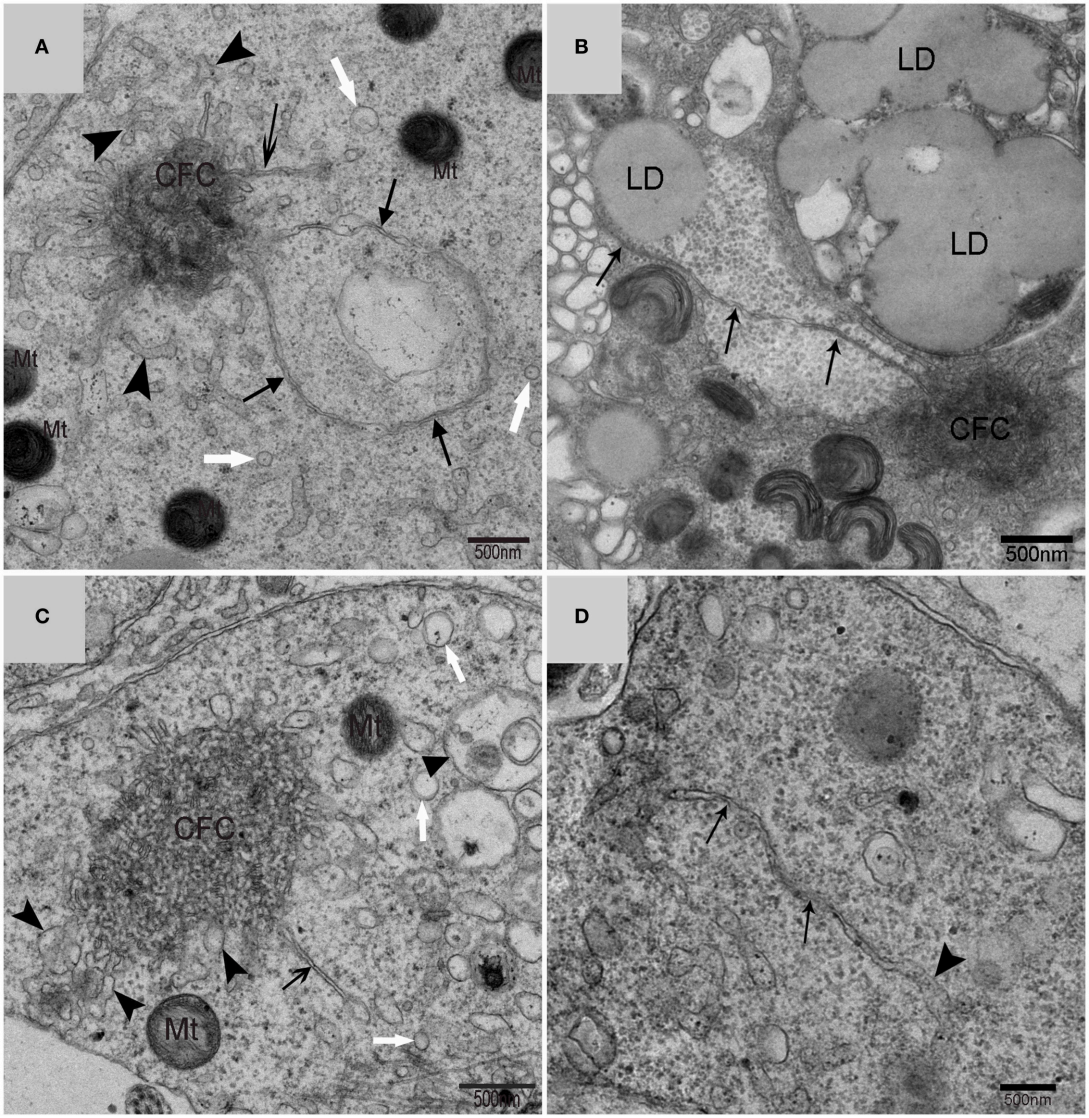
**TEM photograph of the “Chrysanthemum flower center” in the spermatid**. Chrysanthemum flower center (CFC), mitochondrion (Mt), lipid droplet (LD), endoplasmic reticulum (‡), vesicle (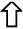), isolation membrane (↑), wrapping autophagosome (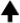), multivesicular body (▴). **(A,B)** Wrapping autophagosome derived from the “Chrysanthemum flower centre” (CFC); **(C)** a vesicle expanded from the end of extending isolation membrane (IM); **(D)** some IM transformed from a single ER.

**Figure 6:**
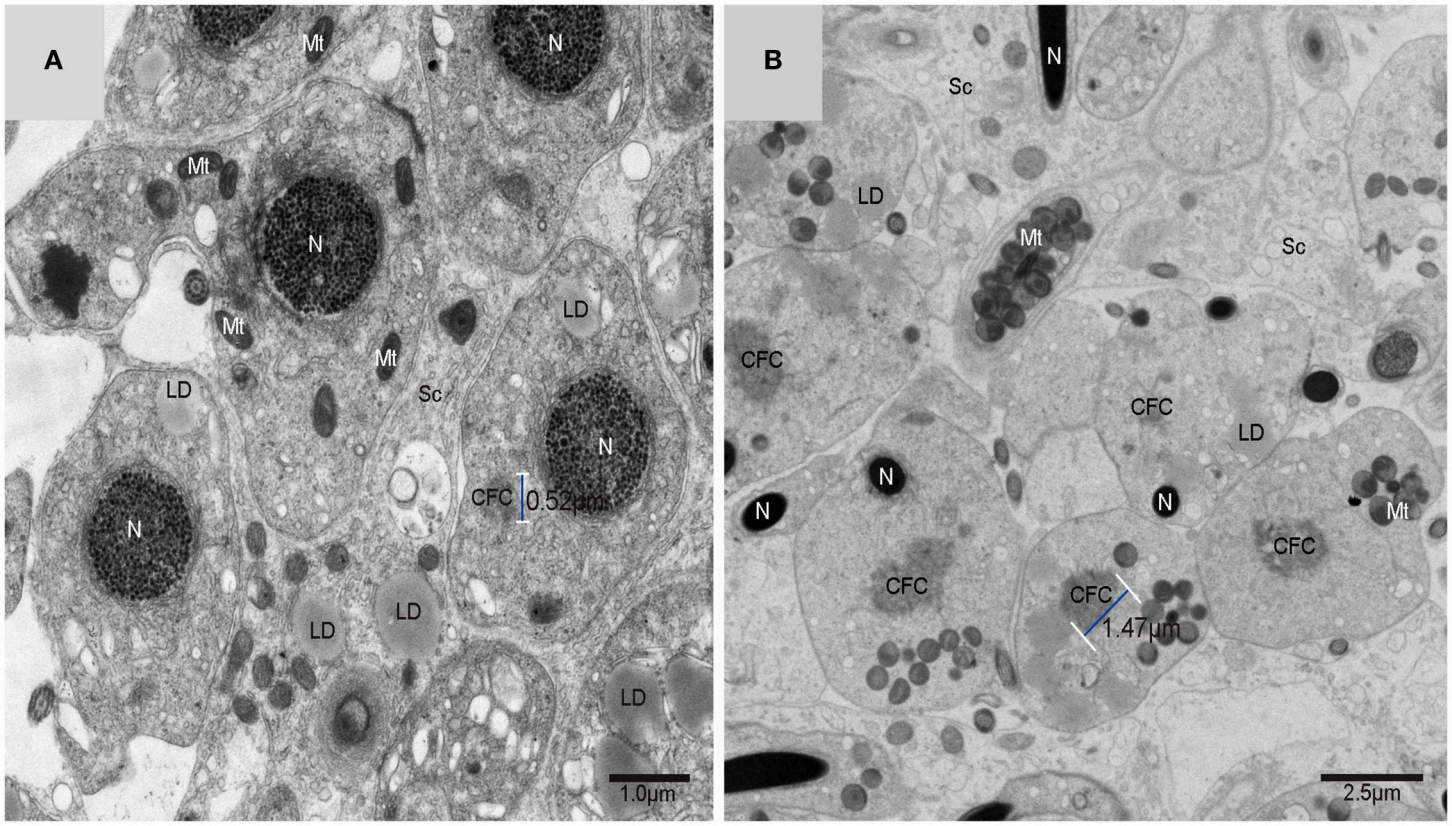
**TEM photograph of spermatids in a sperm column within the testis**. **(A)** Granular nucleus stage (early stage); **(B)** compact nucleus stage (late stage). Nucleus (N), “Chrysanthemum flower center” (CFC), mitochondrion (Mt), Sertoli cell (Sc), lipid droplet (LD).

**Figure 7:**
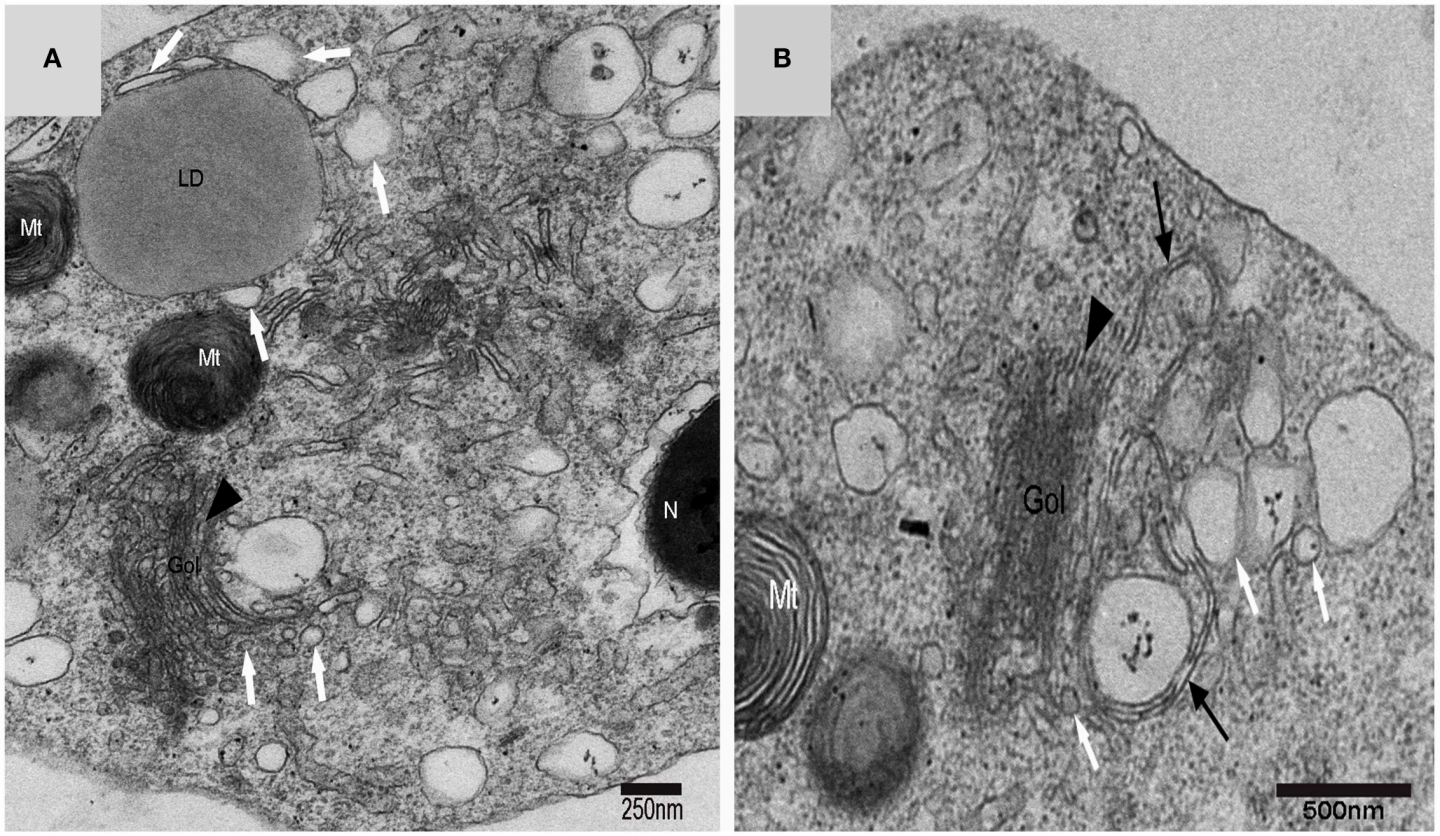
**TEM photograph of a Golgi complex in a spermatid with a compact nucleus**. Golgi complex (Gol), nucleus (N), mitochondrion (Mt), lipid droplet (LD),trans-Golgi network (▴), vesicle from trans face (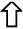), wrapping autophagosome (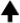). **(A)** Vesicles which clung to lipid droplets; **(B)** a double-layer membrane from the trans-Golgi network.

**Figure 8:**
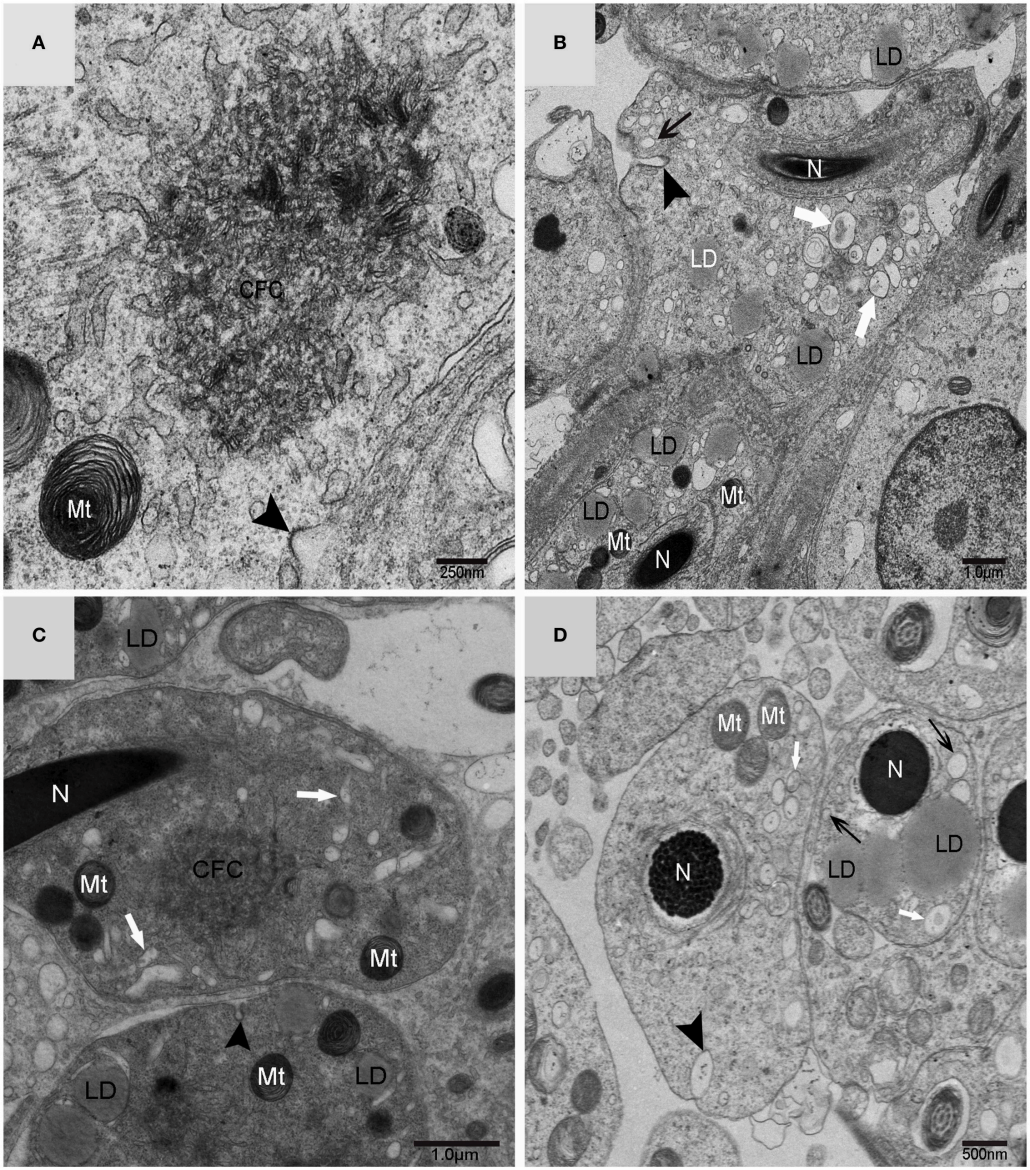
**TEM micrograph of the plasma membrane of the spermatid**. Chrysanthemum flower center (CFC), nucleus (N), mitochondrion (Mt), lipid droplet (LD), plasma membrane invagination (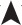), vesicle (↑), endosome (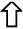). **(A–D)** Some different vesicles which formed by plasma membrane invagination.

Using TEM, numerous autophagosomes with different structures were visualized within germ cells at various developing stages (Figures 3–5, 7, 8). Some were autophagosomes, some were small vesicles, and others were multivesicular bodies (MVB). A special structure 1–2 μm in diameter, named the “Chrysanthemum flower center” (CFC) in this study, was found inside differentiating spermatids (Figures 4–6). Numerous branched ER were distributed around the CFC (Figures 4, 5), and the ER ends inserted into the CFC through short narrow tubules that formed many daisy petal-like structures. These structures comprised the CFC from different directions (Figures 4C,D, 5A), which resembled a chrysanthemum flower. The density of the IM and the short tubular membranes within the CFC was higher than the original ER (Figure 4). Several double layer isolation membranes (IMs) were developed from the CFC (Figures 4, 5), which could elongate and enwrap some cytoplasm along with different structures (Figures 4A, 5A,B). The end of the extending IM expanded sometimes to form a vesicle (Figure 5C) that was similar to some vacuoles inside the spermatid. From late spring to late autumn, the average diameter of the CFC increased from 0.48 ± 0.02 μm to 1.64 ± 0.05 μm (mean ± SE; *n* = 50). The CFC was more developed in spermatids with compact nuclei (Figure 6B) than spermatids with granular nuclei (Figure 6A), which corresponds to late stage and early stage spermiogenesis, respectively. Some IM could also be transformed from a single ER (Figure 5D).

### Vesicles from golgi complexes, plasma membranes and endosomes might also be the sources of autophagosome membrane

On the trans face of the Golgi complex of differentiating spermatids, a large clear vesicle was formed that clung to lipid droplets and became flat (Figure 7A). A double-layer membrane from the trans-Golgi network wrapped some cytoplasm and its vesicles (Figure 7B). Many vesicles in different sizes were distributed throughout the differentiating spermatids (Figure 5C) and some spermatids nestled up against the lipid droplets and became flat (Figure 7A). Plasma membrane invagination was frequently observed (Figure 8), which led to the formation of some different vesicles (Figures 8B,D).

**Figure 9:**
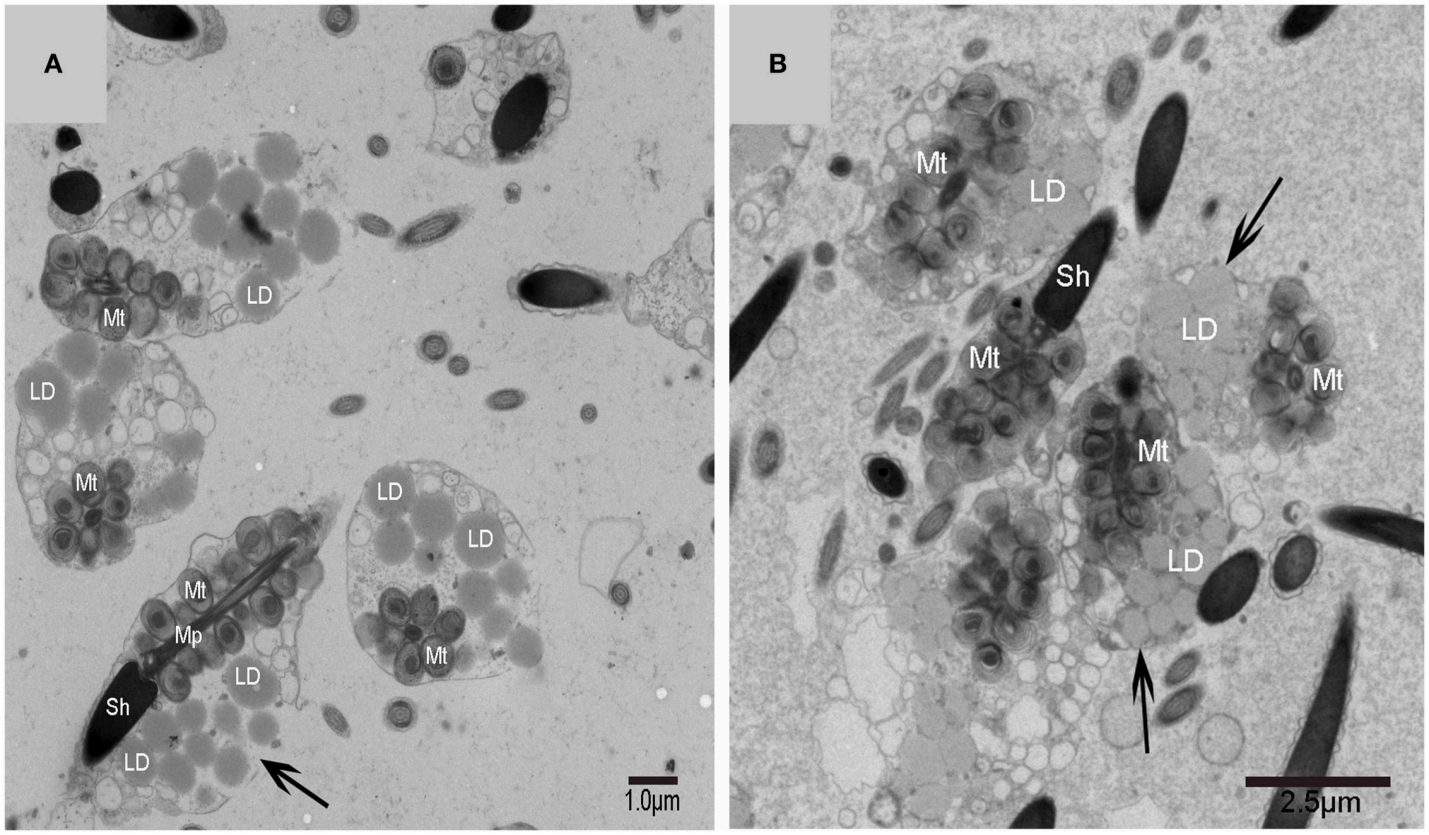
**TEM photograph of mature spermatozoa with cytoplasmic droplet and lipid droplets after spermiation**. Sperm head (Sh), midpiece (Mp), mitochondrion (Mt), lipid droplet (LD), cytoplasmic droplet(↑). **(A,B)** The mature spermatozoon after spermiation.

A very large cytoplasmic droplet with lipid droplets was attached to the midpiece and sperm head of the mature spermatozoon after spermiation in the Chinese soft-shelled turtle (Figure 9). The CFC was no longer observed in mature spermatozoa.

## Discussion

Autophagy plays a vital role in maintaining cellular homeostasis through the continuous turnover of proteins as well as the elimination of defective organelles in lysosomes (Yang and Klionsky, 2010; Bejarano et al., 2014). Unlike other organelles that solidly present in the cell, it is considered that autophagosomes are formed on demand by dynamic membrane rearrangements (Shibutani and Yoshimori, 2014). It is difficult to confirm the membrane sources of autophagosomes because autophagosome formation proceeds rapidly once it is initiated. It is estimated that autophagosome formation takes several minutes in yeast and mammals(Mizushima et al., 2001; Fujita et al., 2008; Geng et al., 2008). Despite tremendous progress made in autophagy, the long-term issues about the source and origin of autophagic membranes remain undiscovered in the past few decades. Recognizing the autophagosome formation site will undoubtedly help to figure out another fascinating question: where does the membrane of autophagosome come from (Hamasaki et al., 2013b)? The testis of the soft-shelled turtle expressed LC3-II, which is the marker protein of the autophagosome, located within spermatids in different stages during spermiogenesis. Numerous autophagosomes were observed inside the spermatids under TEM. A special 1–2 μm diameter structure named the “Chrysanthemum flower center” (CFC) was also found within the spermatid and several double-membrane IM could be produced, elongate and enwrap cytoplasm to develop different autophagosomes from this structure. All the cellular evidence showed that the “Chrysanthemum flower center,” a newly formed structure in the spermatid, may be one important site for IM development and autophagosome formation during cell-size reduction in spermiogenesis. The CFC was more developed in spermatids with compact nuclei than in spermatids with granular nuclei, which corresponds to the late stage and early stage of spermiogenesis, respectively. In late spermiogenesis, the differentiation and remodeling of spermatids into spermatozoa is more active. As far as we know, this study has yielded the first subcellular evidence for an *in vivo* origin and source of IM and autophagosome formation in higher eukaryotes.

In yeast, the membrane of the phagophore and early autophagic structures originate from a single source named the pre-autophagosomal structure (PAS) (Suzuki and Ohsumi, 2010). In higher eukaryotes, phagophore formationmight take place in different positions in the cell, but the current assumption is that phagophores formate at a complex membranous structure named the omegasome. Omegasomes were initially identified as endoplasmic reticulum (ER)-associated spots (Carlsson and Simonsen, 2015). The latest studies have shown that the ER exit sites (ERES), which are specialized ER regions where long-lived proteins are classified into the secretory system, are critical elements in the formation of isolation membranes. Therefore, ERES are a new component that should be concluded into the depiction of autophagosome biogenesis (Sanchez-Wandelmer et al., 2015). It is hypothesized that further research on ERES function will provide provoking insights into eukaryotic cell biology. Electron tomography showed that endoplasmic reticulum associates with isolation membranes or phagophore in mammalian cells. The ER-IM complexs served as a subregion of ER by forming a cradle girding the IM, and showed that both IMs and ER are connected. However, there is little ultrastructural information on the initial stage of autophagosome formation (Hayashi-Nishino et al., 2010). Through TEM, this study demonstrated that the developed Chrysanthemum flower center occurred in differentiating spermatids *in vivo* during turtle spermiogenesis. It was further revealed that CFC could produce long IM that enwrapped some cytoplasm to form the autophagosome. As a result, CFC in the spermatid might be equivalent to a phagophore nucleation site, or the omegasome, or the ER-IM complex in cultured mammalian cells.

Light microscopy experiments provide evidences to demonstrate the continuity between the ER and IMs. In addition, the experiments also found that DFCP1, an ER-resident protein, has access to IMs. (Axe et al., 2008). The mechanism by which the isolation membranes keep their unique identity while remaining connected to their donor membranes remains unclear (Kraft and Martens, 2012). Several pieces of evidence have shown a strong relationship between ER and autophagosome formation sites in mammalian culture cells(Hamasaki et al., 2013b). It was found that 70% of IMs were associated with ER (named ER–IM complexes) in Atg4B (Atg4BC74A) mutant cells, which suggests that the ER–IM complexes are an important early stage of AP formation (Hamasaki and Yoshimori, 2010). As a result, ER is probably the major donor of lipid bilayers for forming autophagosome and omegasome, which could represent the means to supply lipids from ER to a growing phagophore (Sanchez-Wandelmer et al., 2015). A large number of branched ER were distributed around the CFC, and their ends inserted into the CFC through short narrow tubules that formed many daisy petal-like structures that comprised the CFC from different orientations. The morphological connection of narrow tubules showed that the CFC unambiguously came from ER, which could develop IM and form autophagosome in the turtle spermatids. Our data could support the third hypothesis of autophagosome biogenesis that autophagosomes are formed neither by de novo membrane formation nor by direct utilization of ER cisternae (Hayashi-Nishino et al., 2009).

Different biogenesis pathways might regulate autophagy concurrently *in vivo*. Membrane fusion events would ask several autophagic biogenesis complexes to work in a few locations at different stages and could be tied together with diverse membranes. Distinct membrane sources will conduce to biogenesis because with cell's third dimension, many membranes and organelles are close to the phagophore (Abada and Elazar, 2014). It is likely that the Golgi route contributes to autophagosome biogenesis in yeast, although it is not clear if this pathway is important in mammalian systems (Rubinsztein et al., 2012). By tracking Atg9 in Atg9 over-expressing mammalian cells using immune-electron microscopy(IEM), researchers noticed that upon induction of autophagy, a post-Golgi tubulo-vesicular compartment responsive for Atg9 transfers to the vacuole(Mari et al., 2010). Furthermore, the tubulo-vesicular compartment experiences homotypic fusion and reestablishment for purpose of producing isolation membranes (IMs) (Kraft and Martens, 2012). How vesicular precursors integrate into IMs is still unknown. In cultured mammalian cells, Atg9 partly colocalizes to trans-Golgi network and endosomes (Hamasaki et al., 2013b). Additionally, depriving of nutrition induces Atg9 vesicles exit from the trans-Golgi network (TGN) and instantaneous co-localization of Atg9 with LC3 (the autophagosome marker) in fluorescence microscopy experiments. (Young et al., 2006; Orsi et al., 2012). Hamasaki and Yoshimori revealed that Atg9 localization intensively suggests the trans-Golgi network (TGN) and endosome as possible origins of autophagosomal membranes (Hamasaki and Yoshimori, 2010). Using cytotoxic stressors, another study suggested the trans-Golgi network (TGN) as a potential membrane origin in Atg5/Atg7-independent alternative autophagy (Nishida et al., 2009). Double-layer membranes from the trans-Golgi network could wrap some cytoplasm and its vesicles to form autophagosomes in turtle spermatids, which suggests that Golgi complex might be another important autophagy biogenesis site in addition to the CFC. On the trans-face of the Golgi complex of the differentiating spermatids, a large clear vesicle was formed. These vesicles could became flat and cling to the lipid droplet, which bore the characteristics of lipophagy (Liu and Czaja, 2013; Dupont et al., 2014).

Experiments revealed that membrane traffic from early to recycling endosomes is crucial for autophagosome formation and that the recycling compartment is not merely responsible for recycling of plasma membrane receptors but also serves as a station in the early stages of autophagosome biogenesis (Puri et al., 2013). It has been proposed that isolation membranes are formed by the fusion of vesicular carriers that are derived from the plasma membrane (Ravikumar et al., 2010; Moreau et al., 2011) or the Golgi apparatus (Ohashi and Munro, 2010; van der Vaart et al., 2010). Owing to autophagosome formation occurring away from donor membranes, diffusion barrier has no use for separating isolation membranes (IMs) from donor membranes in the vesicular fusion models (Kraft and Martens, 2012). Researchers revealed that the plasma membrane makes contributions to autophagosome formation and it is particularly significant in time of increased autophagy (Rubinsztein et al., 2012). Plasma membrane invagination was frequently observed on the spermatid membrane, which could lead to the formation of different vesicles and endosomes distributed around the CFC and autophagosomes or distribution close to the lipid droplet such as the Golgi vesicle in present study. The size reduced sharply from spermatids to spermatozoa. The plasma membrane's large surface area might act as a massive membrane store that allows cells to experiences cycles of autophagosome synthesis at much higher rates than under basic conditions. And it does not compromise other processes. Recent studies demonstrate the idea that these autophagy-related proteins and membranes that are necessary for autophagosome formation are originated from plasma membrane and that these components coalesce in recycling endosomes that are an early PAS or phagophore intermediate (Sanchez-Wandelmer et al., 2015).

**Figure 10:**
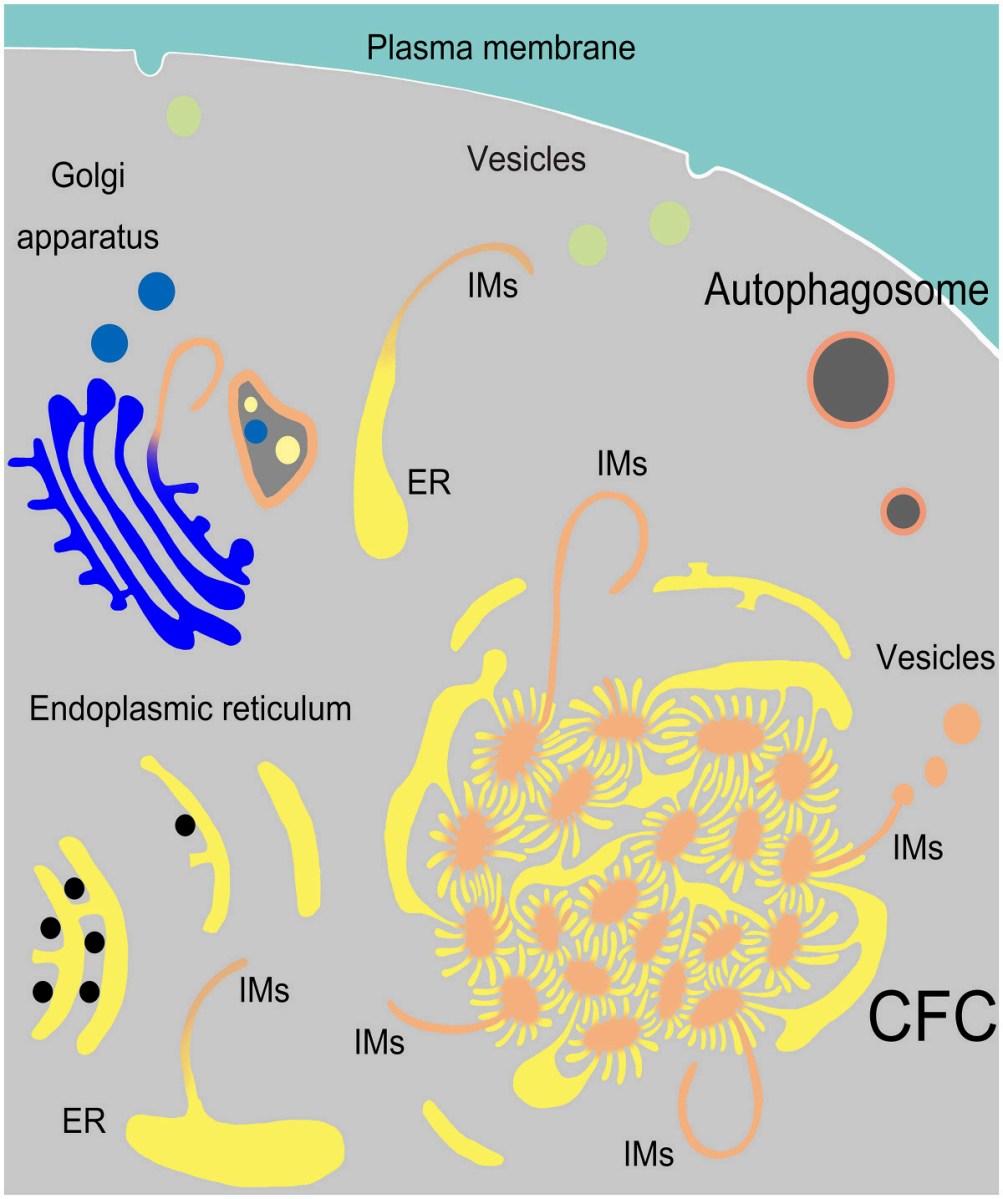
**Model of IM biogenesis during spermatogenesis in Chinese soft-shelled turtle**. Many endoplasmic reticula (ER) were transferred into a special “Chrysanthemum flower center” (CFC), from which several double-layer isolation membranes (IM) were formed and extended. Narrow tubules connected the ends of ER and the CFC. An IM could also be transformed from a single ER. Sometimes IM extended from a trans-Golgi network and wrapped different structures.

In the present study, we determined that endoplasmic reticulum, vesicles from Golgi complexes, plasma membranes and endosomes might be the sources of autophagosomal membrane (Figure 10). The discovery of ER-derived “Chrysanthemum flower center” (CFC) supports the possibility of the cradle/omegasome model (Hamasaki and Yoshimori, 2010) or de novo membrane formation (Hayashi-Nishino et al., 2009). This finding brings us closer to understanding the origin of autophagosomal membranes. However, details in the formation or constituents of CFC still need further investigation.

## Author contributions

The authors have made the following declarations about their contributions: YH and QC Conceived and designed the experiments. YH, HC, TL, XC, QL, YL, LH, and QZ Performed the experiments. PY, TL, and NA analyzed the data. YH and QC Wrote the paper. All authors read and approved the final manuscript.

## Funding

This research was supported by grants from the National Natural Science Foundation of China (Grant number: 31272521 and 31672505) and Priority Academic Program Development of Jiangsu Higher Education Institutions, China.

### Conflict of interest statement

The authors declare that the research was conducted in the absence of any commercial or financial relationships that could be construed as a potential conflict of interest.
